# ELABELA/APJ Axis Prevents Diabetic Glomerular Endothelial Injury by Regulating AMPK/NLRP3 Pathway

**DOI:** 10.1007/s10753-023-01882-7

**Published:** 2023-08-04

**Authors:** Zhida Chen, Zhe Wang, Yepeng Hu, Huangbo Lin, Li Yin, Jing Kong, Yikai Zhang, Bibi Hu, Tiekun Li, Xianan Zheng, Qiongying Yang, Shu Ye, Shengyao Wang, Qiao Zhou, Chao Zheng

**Affiliations:** 1 Department of Nephrology, The Second Affiliated Hospital, School of Medicine, Zhejiang University, Hangzhou, China; 2 Department of Endocrinology, The Second Affiliated Hospital, School of Medicine, Zhejiang University, Hangzhou, China; 3 School of Medical Information Engineering, Guangzhou University of Chinese Medicine, Guangzhou, China; 4 Department of Vascular Surgery, The Second Affiliated Hospital, School of Medicine, Zhejiang University, Hangzhou, China; 5 Nanjing Kingmed Center for Clinical Laboratory, Nanjing, China

**Keywords:** ELA, Glomerular endothelial injury, Diabetic kidney disease, NLRP3, AMPK

## Abstract

ELABELA (ELA), a recently discovered peptide, is highly expressed in adult kidneys and the endothelium system. It has been identified as a novel endogenous ligand for the apelin receptor (APJ). This study aims to investigate the role of ELA in diabetic glomerular endothelial pyroptosis and its underlying mechanism. Initially, a significant decrease in ELA mRNA levels was observed in the renal cortex of *db/db* mice and high glucose–treated glomerular endothelial cells (GECs). It was also found that ELA deficiency in ELA^+/−^ mice significantly accelerated diabetic glomerular injury, as shown by exacerbated glomerular morphological damage, increased serum creatine and blood urea nitrogen, and elevated 24-h urinary albumin excretion. In addition, *in vivo* overexpression of ELA prevented diabetic glomerular injury, reduced von Willebrand factor expression, restored endothelial marker CD31 expression, and attenuated the production of adhesive molecules such as intercellular adhesion molecule-1 and vascular cell adhesion molecule-1. Furthermore, *in vitro* studies confirmed that treatment with ELA inhibited GEC injury by regulating the NOD-like receptor protein 3 (NLRP3) inflammasome, as indicated by blocking NLRP3 inflammasome formation, decreasing cleaved Caspase-1 production, and inhibiting interleukin-1β and interleukin-18 production. Moreover, *in vitro* experiments demonstrated that the protective effects of ELA in GECs during hyperglycemia were diminished by inhibiting adenosine monophosphate–activated protein kinase (AMPK) using Compound C or by APJ deficiency. Taken together, this study provides the first evidence that ELA treatment could prevent diabetic glomerular endothelial injury, which is partly mediated by the regulation of the AMPK/NLRP3 signaling pathway. Therefore, pharmacologically targeting ELA may serve as a novel therapeutic strategy for diabetic kidney disease.

**Supplementary Information:**

The online version contains supplementary material available at 10.1007/s10753-023-01882-7.

## INTRODUCTION

Diabetic kidney disease (DKD) represents one of the most common microvascular complications of both type 1 and type 2 diabetes mellitus, which is the leading cause of end-stage renal disease (ESRD) in China. Despite efforts to control serum glucose levels, manage lipid levels, inhibit the renin-angiotensin system (RAS), and utilize sodium-glucose co-transporter 2 inhibitors (SGLT2is) and mineralocorticoid receptor antagonist (MRA), the progression of DKD cannot be entirely halted, leading many patients to eventually develop ESRD [1–5]. Thus, there is an urgent need to explore the underlying mechanisms and discover novel approaches to cure

Recent evidence strongly indicates that glomerular endothelial cell (GEC) injury plays a significant role in the pathological development of microalbuminuria and glomerular sclerosis [6]. As the first layer of the glomerular filtration barrier, endothelial cells are highly responsive to changes in serum glucose levels. Hyperglycemia not only increases the permeability of glomerular endothelial cells and induces endothelial cell apoptosis, but also exacerbates the excretion of cytokines and inflammatory molecules [7].

Although there are various molecular mechanisms underlying hyperglycemia-induced GEC dysfunction, inflammasome activation plays a crucial role in the early stages of DKD and contributes to progressive renal function damage [8]. Among the inflammasomes, the NOD-like receptor protein 3 (NLRP3) inflammasome is the most well-demonstrated. Upon hyperglycemic stimulation, NLRP3 recruits and assembles with apoptosis-associated speck-like protein containing a CARD (ASC) and Caspase-1, resulting in the formation of the NLRP3 inflammasome complex. Subsequently, NLRP3 cleaves pro-Caspase-1 into its active form, known as cleaved Caspase-1, which is essential for the maturation of IL-1β and IL-18, which then promotes immune cell recruitment [9]. Additionally, adhesive molecules such as intercellular adhesion molecule-1 (ICAM-1) and vascular cell adhesion molecule-1 (VCAM-1) can be upregulated in response to IL-1β and IL-18, which facilitate the transmigration of lymphocytes and contribute to renal inflammatory damage [10]. Based on these findings, targeting the inflammatory process in GECs may represent a viable strategy for the treatment of DKD.

Intriguingly, there has been growing interest in identifying potential regulators that can mediate both metabolic processes and inflammatory pathways. Previous studies have highlighted adenosine monophosphate–activated protein kinase (AMPK) as a crucial cellular energy sensor, with its activity showing an inverse relationship with increased inflammatory markers in obese individuals [11]. For instance, the AMPK activator AICAR has been shown to alleviate NLRP3 inflammasome activation and subsequent production of IL-1β induced by palmitic acid [12]. Moreover, metformin, a widely used medication for type 2 diabetes, has been found to protect against myocardial ischemia–reperfusion injury by regulating the AMPK/NLRP3 inflammasome pathway [13]. Furthermore, the dipeptidyl peptidase-4 (DPP-4) inhibitor omarigliptin has been shown to diminish NLRP3 inflammasome formation and excessive production of IL-18 and IL-1β through AMPK activation in glucose-stressed GECs [14]. Thus, AMPK represents a promising candidate for co-regulating metabolic disease and inflammasome activation, making it a potential therapeutic target for inflammatory processes [15].

The apelin system is an endogenous physiological regulator in various diseases and consists of the apelin receptor (APJ) and its two endogenous ligands, apelin and ELABELA (ELA). ELA, the second ligand of APJ, is predominantly found in adult kidneys and the endothelium system [16]. Recent studies have revealed that ELA enhances cardiac contractility, promotes vasodilation, regulates fluid balance, and possesses anti-atherosclerotic and anti-oxidative properties through its interaction with the apelin receptor [17, 18]. Moreover, ELA gene therapy was reported to preserve glomerular architecture, attenuate renal fibrosis, and suppress the expression of fibrotic molecules in the kidneys of hypertensive rodent models [19, 20]. Additionally, gene therapy involving ELA was reported to improve endothelial cell function via the ELA-APJ axis in human umbilical vein endothelial cell (HUVEC) lines [21].

Despite these revelations, it remains unclear whether ELA has beneficial effects on diabetic models, and the underlying mechanisms require further investigation. Previous studies in our laboratory have shown that ELA exhibits anti-inflammatory and anti-fibrotic effects in DOCA/salt-induced hypertensive nephropathy by inhibiting the formation and activation of the NLRP3 inflammasome [20]. Based on these findings, we believe it is of great interest to investigate whether ELA could also exert beneficial effects on DKD, particularly by targeting the inflammatory processes occurring in GECs. Therefore, the present study was designed to investigate the role of ELA in diabetic glomerular endothelial cells and the potential molecular mechanism involved.

## METHODS

### Animals and Experiment Protocol

All the experiments were approved by the Animal Care Committee of Zhejiang Chinese Medical University. Heterozygous ELA knockout C57BL/6 (ELA^+/−^) mice, wild-type C57BL/6 mice, *db/db* mice on the C57BL/KsJ background, and *db/m* littermates were used in this study. The ELA heterozygous and wild-type C57BL/6 mice were provided by GemPharmatech (Nanjing, China). The genotyping figure of heterozygous ELA knockout mice is shown in Supplementary Fig. 1. The primer information was listed below. 5′arm F1: CCAGGGAGTCTTTCTGTCTCCACT, R1: CTCGGGGTGATTCATATCCAGC; 3′arm F2: AGAAAATCAAGCTGTGGGAGGATG, R2: CACTGGCCTTTTAGTGTAAGAACGC. The *db/db* mice and *db/m* mice were purchased from the Animal Model Research Center of Nanjing University. All the mice were granted free access to water and received normal-chow diet, under standard laboratory conditions with controlled temperature (22–25 °C), humidity (60%), and 12-h light/dark cycle conditions.

The 6–8-week ELA^+/−^ heterozygous and wild-type C57BL/6 mice were divided into three groups: control + wild-type group, STZ + wild-type group, and STZ + ELA^+/−^ group. After 1 week of adaptive feeding, uninephrectomy was performed to accelerate the development of DKD, as previously described [22]. Then, after a 1-week recovery period, diabetes was induced in mice by intraperitoneal injection of streptozotocin (STZ) at a dose of 50 mg/kg for three consecutive days. In contrast, mice in the control group received an equivalent volume of citrate buffer. On day 7, mice with fasting blood glucose > 12 mmol/L were considered diabetic and included in the study for further analysis [22, 23].

Next, 6–8-week *db/db* mice were randomly divided into control mice and ELA-overexpression mice (*n* = 5/group). Similarly, the *db/m* mice were also randomly divided into control mice and ELA-overexpression mice groups (*n* = 5/group). Then, intrarenal transfection was performed as previously described [24, 25]. Briefly, the lentivirus expressing elabela (his-ELA-lv) and negative controls (NC-lv) were produced by Gene Pharma (Shanghai, China). After a week of adaptation, both *db/m* and *db/db* mice were anesthetized with inhaled isoflurane. Once fully anesthetized, a median ventral incision was made to expose the kidneys and was exposed and injected in three sites with his-ELA-lv or NC-lv (2 × 10^9^ IU/kidney) dilution with 100 μl sterile 0.9% sodium chloride solution. Studies had shown that lentiviral-mediated protein expression in kidney parenchyma is significantly upregulated within 2 days and maintained for more than 2 months [24].

During the experiments, the blood glucose levels and body weight of the mice were measured weekly. For sample collection, the mice were anesthetized with 2% isoflurane, and blood samples were collected from the heart to measure various parameters, including serum glucose, serum creatinine, serum blood urea nitrogen (BUN), urine glucose, and 24-h urinary albumin, using a fully automatic biochemical analyzer (Hitachi, Japan).

### Morphological and Immunohistochemical Analysis

Tissue sections were subjected to periodic acid-Schiff (PAS) staining for morphological analysis. The glomerular damage index (GDI) was evaluated by two independent examiners blinded to the animal groups. GDI scoring was based on the extent of glomerular damage, as previously described [26]. The severity of lesions was graded on a scale of 0 to 4, depending on the percentage of glomerular involvement. The scoring criteria were as follows: 0 = no damage,1 = mild damage (less than 25% of the glomerular area affected); 2 = moderate damage (25–50% of the glomerular area affected); 3 = intermediate damage (50–75% of the glomerular area affected); and 4 = severe damage (more than 75% of the glomerular area affected). The average scores from multiple counted glomeruli were used to calculate the glomerular damage index for each animal. Immunohistochemical analysis was conducted as previously described [20]. The primary antibodies were ICAM-1 (1:200, Abcam) and VCAM-1 (1:200, Abways).

### Immunofluorescence

Frozen kidney tissue sections with a thickness of 7 μm were fixed using acetone and permeabilized with 0.1% Triton X-100. Subsequently, the tissue sections were incubated overnight at 4 °C with the following primary antibodies: NLRP3 (1:200, Abcam), Caspase-1 (1:200, Santa Cruz), CD31 (1:100, Abcam), von Willebrand factor (vWF) (1:100, Abcam). After incubation, the tissue sections were washed and labeled with corresponding secondary antibodies conjugated to Alexa Fluor-488 (1:500, Invitrogen) and Alexa Fluor-555 (1:500, CST) for 1 h at room temperature. After further washing, the tissue sections were observed using a confocal laser scanning microscope (Fluoview FV1000, Olympus, Japan). Image Pro Plus software (version 6.0; Media Cybernetics, Bethesda, MD) was used to analyze colocalization and quantified as the Pearson correlation coefficient.

### Cell Culture

The rat GECs used in this study were obtained from Procell Life Science & Technology Company, Wuhan, China (CP-R063). The GECs were isolated and cloned, as previously reported [27]. Briefly, the renal cortex was cut into 1 to 2 mm^3^ thick fragments and filtered through 150- and 200-mesh steel sieves. Glomeruli collected in the 200-mesh steel sieve were further processed. After washing with serum-free DMEM, the glomeruli were seeded in a culture flask pre-coated with 10 mg/ml collagen I and a prepared endothelial cell medium. The GECs were grown into a confluent monolayer during the second week and were frozen for later use at passage 3 or 4. The characteristics of the GECs were kept such as positive staining with CD31 [27]. The identification of rat GECs is shown in Supplementary Fig. 1B. The GECs were cultured in Dulbecco’s Modified Eagle’s Medium (DMEM, Gibco) supplemented with 10% fetal bovine serum (Gibco) and 1% antibiotics (100 IU/ml penicillin–streptomycin) at 37 °C in a 5% CO_2_ atmosphere until used for further experiments.

Next, the GECs were pretreated with ELA peptide (1 μmol/l, Phoenix Pharmaceuticals) for 1 h, as previously described [28, 29], followed by treatment with high glucose (HG, 30 mmol/l, Sigma). In the mechanical studies, the cells were pretreated with Compound C (20 mmol/l, MCE), A-769662 (125 μmol/l, Abcam), and AICAR (100 μmol/l, MCE) for 1 h, then stimulated with high glucose for 24 h.

### The shRNA Interference Technology and Small Interfering RNA (siRNA) Transfection

The shRNA interference technology was used to inhibit APJ expression in GECs. The GECs were infected with lentivirus containing specific shRNA against APJ (target sequence (GCTGATATCTTCATTGCTAGC) or scrambled shRNA sequence (TTCTCCGAACGTGTCACGT) as negative control (GenePharma, Shanghai, China) for 12 h prior to treatment with HG for additional 24 h.

AMPK siRNA and control siRNA were purchased from GenePharma. The glomerular endothelial cells were transfected with control siRNA or AMPK siRNA duplex using Lipofectamine 2000 (Invitrogen). Cells were harvested after 48 h transfection for further experiments. Control sense: UUCUCCGAACGUGUCACG UTT; siAMPK sense: GUGGCAGUUAAGAUCUUA ATT.

### RNA Extraction and Quantitative Real-Time PCR

Quantitative real-time PCR was performed as previously described [30]. The primers were designed and synthesized by Tsingke Biotech Com (Shanghai, China). The primer sets used in this study are shown in Supplementary Table 1.

### Western Blot Analysis

Western blot was performed as described previously [20]. Briefly, the samples were incubated with primary antibodies, including ICAM-1 (1:1000, Abways), VCAM-1 (1:1000, Abways), cleaved Caspase-1 (1:1000, Santa Cruz), NLRP3 (1:1000, Abways), p-AMPK (1:1000, CST), AMPK (1:1000, Affinity), APJ (1:1000, Proteintech) and β-actin (1:2000, Abcam) at 4 °C overnight. After washing with Tris-buffered saline, the membranes were incubated with horseradish peroxidase–conjugated secondary antibody (1:5000, Yeasen) at 37 °C for 1 h and visualized using the Amersham Image Quant 800 imaging system (GE Healthcare). The ratios for the examined proteins were normalized to β-actin and are expressed as the means ± standard errors of the mean (SEM).

### ELISA Assay

The ICAM-1 and VCAM-1 levels in the supernatant of GECs were assessed by enzyme-linked immunosorbent assay (MLBio, China) following the manufacturer’s instructions.

### Statistical Analysis

The data obtained are presented as means ± SEM (standard error of the mean). Statistical significance was determined using either a two-tailed Student’s *t*-test or ANOVA (analysis of variance) followed by a Student–Newman–Keuls post hoc test for multiple comparisons. Differences were considered significant for *p* values < 0.05.

## RESULTS

### ELA Deficiency Exacerbated Diabetic Glomerular Injury *In Vivo*

**Fig. 1:**
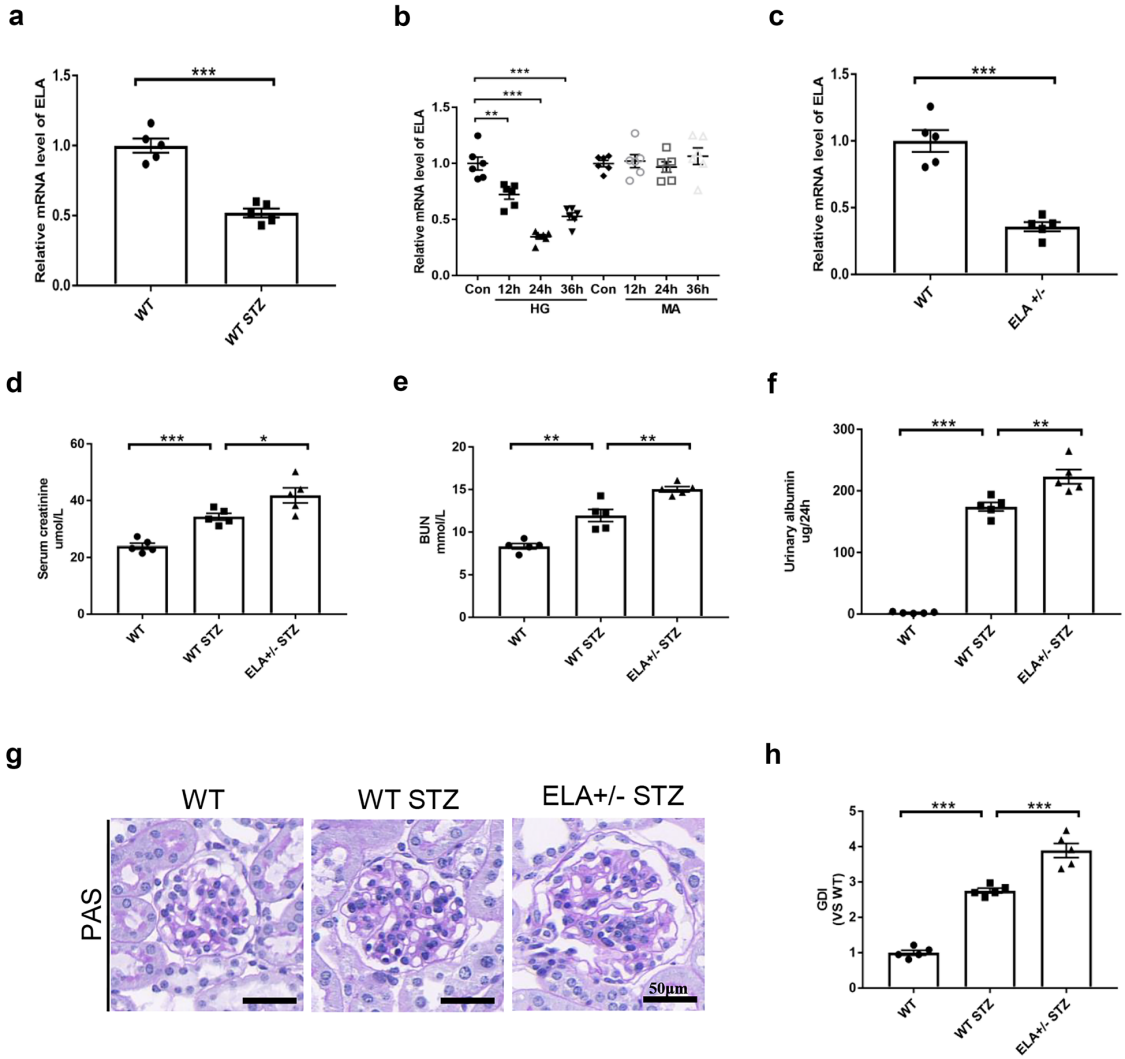
ELA deficiency exacerbates diabetic glomerular injury *in vivo*. **A** ELA mRNA expression in the cortex of STZ-induced mice (*N* = 5). **B** ELA mRNA expression on HG or MA treatment in GECs (*N* = 6). **C** The mRNA level of ELA in the kidney of heterozygous ELA knockdown C57BL/6 mice (ELA^+/−^) compared to WT ones (*N* = 5). *P < 0.05 versus wild-type group. **D**–**F** Serum creatine, serum BUN, and 24-h urinary albumin in wild-type mice and ELA KD mice (*N* = 5). **G** Representative photomicrograph showing glomerular structures (PAS staining) and the summarized glomerular damage index (GDI) in different groups (*N* = 5). *P < 0.05, **P < 0.01, ***P < 0.001.

As shown in Fig. 1A, the mRNA level of ELA was approximately 50% lower in the cortex of mice with STZ-induced diabetes. Next, the mRNA expression of ELA was assessed in glucose-stressed GECs. After HG stimulation, ELA mRNA expression was significantly reduced at all time points examined (12–36 h), with the lowest expression observed at 24 h. Notably, treatment with a high concentration of mannitol (27.5 mM mannitol + 5.5 mM glucose) did not affect the mRNA levels of ELA, suggesting that the effect of HG on ELA expression was not solely due to high osmotic pressure (Fig. 1B). To investigate the role of ELA in the progression of DKD, ELA^+/−^ mice were used to observe renal injury in the STZ-induced model. As shown in Fig. 1C, a successful knockdown of ELA mRNA expression (approximately 65% decrease) was observed. Serum glucose and urinary glucose levels were comparable between STZ-injected ELA^+/−^ mice and wild-type mice, and both groups exhibited similar degrees of weight loss (Fig. S2). However, ELA^+/−^ diabetic mice displayed significantly elevated levels of serum creatinine, serum BUN and 24-h urinary albumin compared to wild-type diabetic mice (Fig. 1D–F). Furthermore, ELA knockdown exacerbated abnormal glomerular damage, characterized by severe capillary collapse, increased mesangial cell expansion, enhanced extracellular matrix accumulation, and collagen deposition (Fig. 1G). The glomerular damage index (GDI) was higher in ELA knockdown diabetic mice compared to wild-type diabetic mice (Fig. 1H).

### ELA Overexpression Decreased Serum Creatinine, Serum BUN, 24-h Urinary Albumin, and Glomerular Endothelial Injury in db/db Mice

**Fig. 2:**
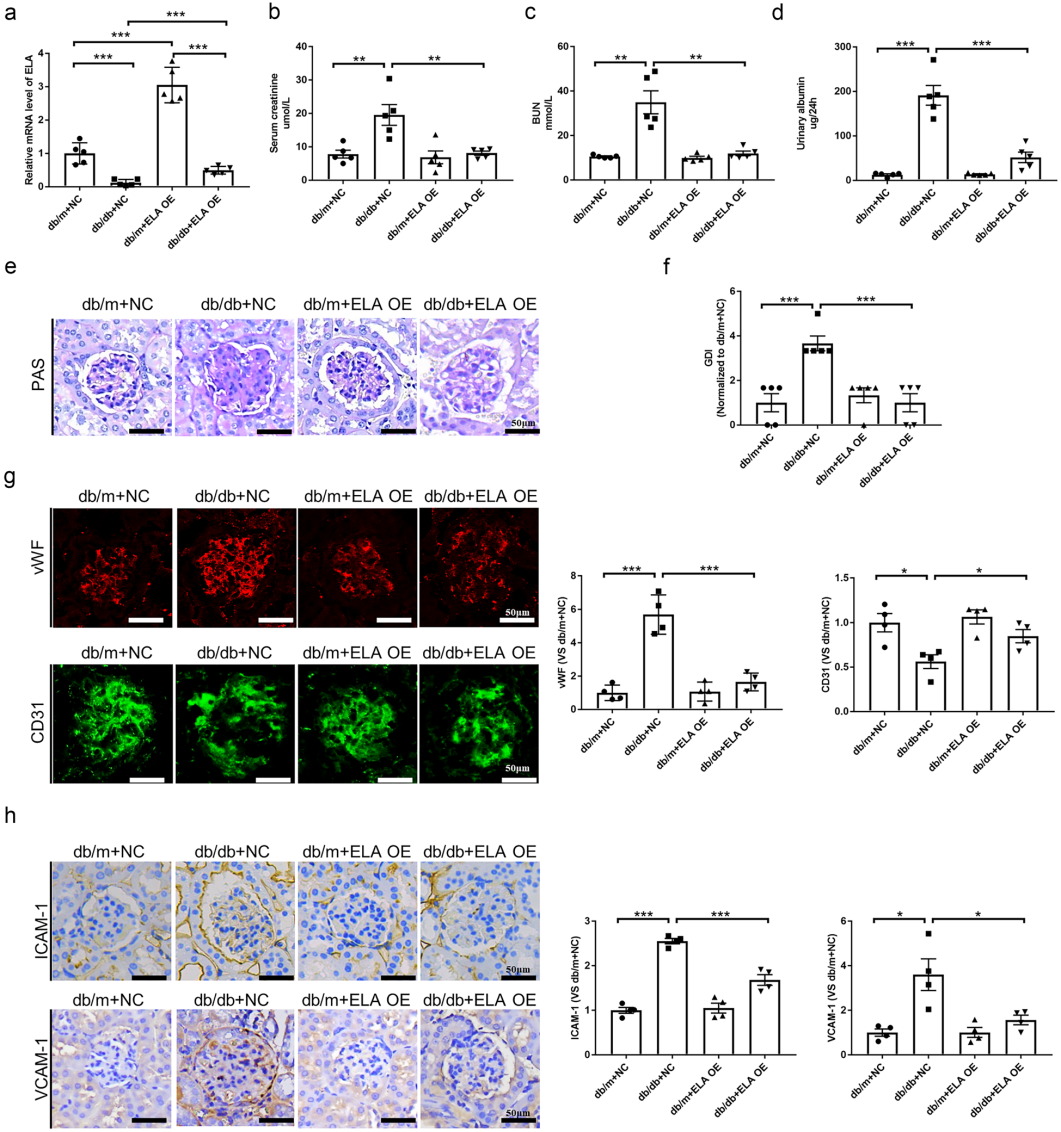
ELA overexpression improves diabetic glomerular injury *in vivo*. **A** The mRNA expression of ELA in the cortex of *db/db* mice and *db/m* mice with or without ELA overexpression (*N* = 5). **B**–**D** Serum creatinine, serum BUN, and 24-h urinary albumin in different groups (*N* = 5). **E**, **F** Representative photomicrograph showing glomerular structures (PAS staining) and the summarized glomerular damage index in different groups (*N* = 5). **G** Immunofluorescence staining of VWF and CD31 in glomeruli and the summarized immunofluorescence intensity (*N* = 4). **H** Representative immunohistochemical staining of ICAM-1 and VCAM-1 in glomeruli and the summarized blot intensities (*N* = 4). Scale bar, 50 μm. *P < 0.05, **P < 0.01, ***P < 0.001.

To further investigate the beneficial role of ELA *in vivo*, we performed intrarenal transfection of ELA lentivirus or NC lentivirus in the renal cortex of different groups. After a 12-week normal diet, the mRNA expression of ELA was assessed in the renal cortex. As depicted in Fig. 2A, the mRNA level of ELA was approximately 80% lower in the cortex of *db/db* mice compared to *db/m* mice. However, after intrarenal transfection of ELA lentivirus, the mRNA expression of ELA was significantly increased in both the *db/m* + NC group and the *db/db* + NC group. Figure S3A–C show that blood glucose levels, urinary glucose levels, and body weight were significantly elevated in *db/db* mice compared to control mice. However, no significant difference was observed in *db/db* mice with or without ELA lentivirus transfection, indicating that ELA did not affect these parameters in the diabetic mice. Furthermore, serum creatinine, serum BUN, and 24-h total urinary albumin levels were significantly increased in untreated *db/db* mice compared to control mice. However, when ELA lentivirus was transfected into *db/db* mice, these increases in serum creatinine, serum BUN, and urinary albumin were alleviated (Fig. 2B–D). Furthermore, the *db/db* mice exhibited abnormal glomerular morphology, characterized by capillary collapse, mesangial cell expansion, extracellular matrix accumulation, and collagen deposition. The GDI was approximately twice as high in *db/db* mice compared to *db/m* mice. In contrast, the deterioration of glomerular morphology was significantly diminished in *db/db* mice after treatment with ELA (Fig. 2E–F). Moreover, the expression of CD31, a classic endothelial marker, was significantly decreased in *db/db* mice, indicating the loss of glomerular capillaries. Additionally, hyperglycemic stress contributed to endothelial injury, as evidenced by a marked increase in the expression of vWF. However, treatment with ELA significantly preserved CD31 expression and alleviated vWF expression in *db/db* mice. Quantification of fluorescence intensity from CD31 and vWF staining is summarized in Fig. 2G. Moreover, the expression of ICAM-1 and VCAM-1 was markedly increased in the glomeruli of *db/db* mice compared to control mice, suggesting an enhanced adhesive function in the glomeruli of *db/db* mice. However, ELA overexpression alleviated the expression of ICAM-1 and VCAM-1 in the glomeruli of diabetic mice (Fig. 2H).

### ELA Overexpression Prevented NLRP3 Inflammasome Activation in GECs of db/db Mice

**Fig. 3:**
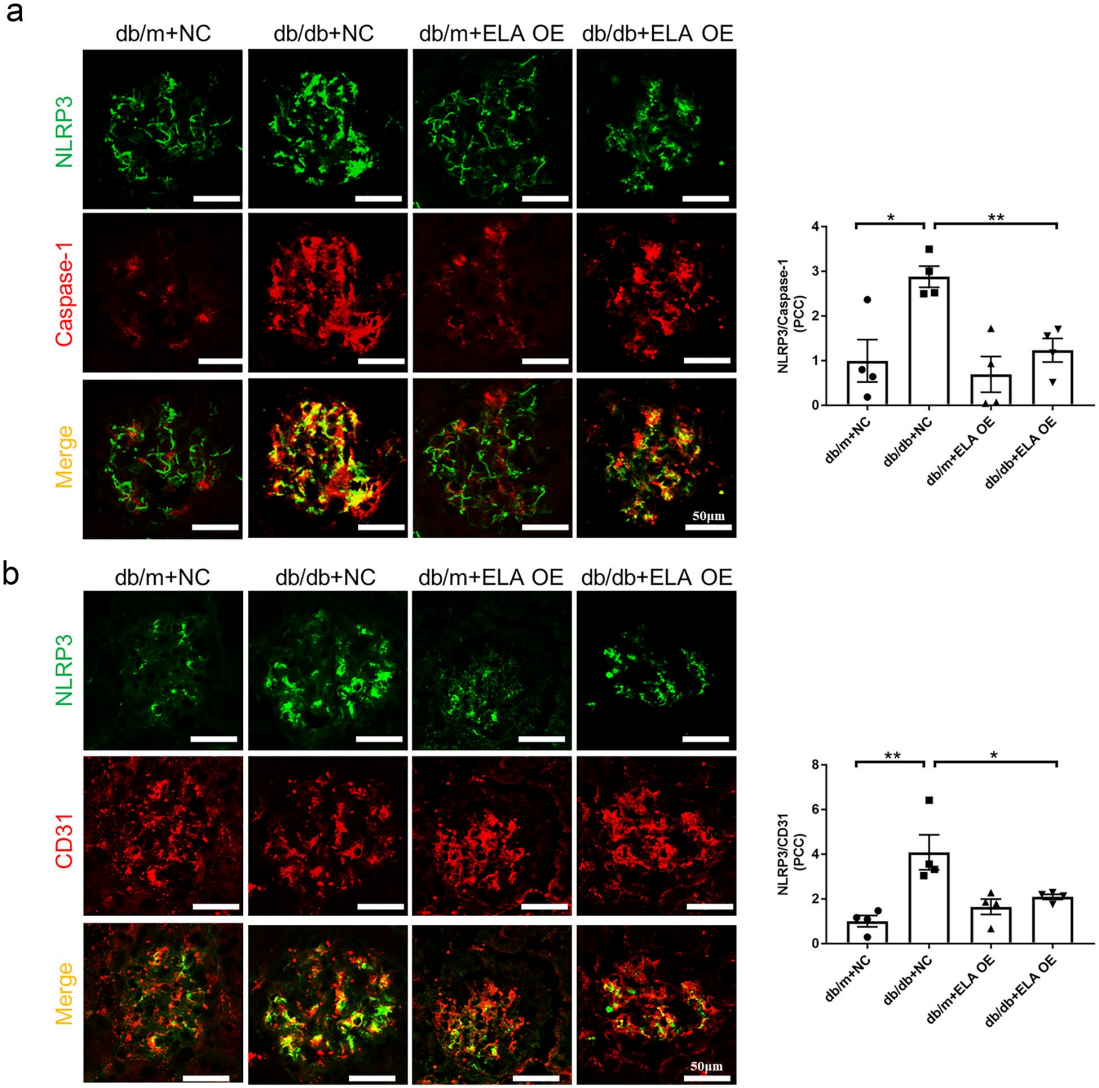
ELA overexpression inhibits NLRP3 inflammasome formation in GECs *in vivo*. **A** Representative confocal fluorescent images of colocalization (yellow) between NLRP3 (green) and Caspase-1 (red) and the summarized colocalization coefficient in glomeruli (*N* = 4). **B** Representative confocal fluorescent images of colocalization (yellow) between NLRP3 (green) and CD31 (red) in the glomeruli and the summarized colocalization coefficient in glomeruli (*N* = 4). Scale bar, 50 μm. *P < 0.05, **P < 0.01.

Previous studies conducted in our laboratory demonstrated that ELA treatment suppressed NLRP3 inflammasome formation and improved inflammatory responses in aldosterone-induced HK2 cells [20]. To investigate the potential anti-inflammatory role of ELA in GECs, we first examined whether NLRP3 inflammasome formation and activation were involved in diabetes-induced GECs *in vivo*. As shown in Fig. 3A, hyperglycemia significantly increased the colocalization of NLRP3 with Caspase-1 in the glomeruli of *db/db* mice compared to control mice, indicating the formation of NLRP3 inflammasomes in diabetic mice. However, the administration of ELA substantially blocked NLRP3 inflammasome formation in the glomeruli. The quantification of NLRP3 colocalization was determined using Pearson’s correlation coefficient. Furthermore, while the expression of the endothelial marker CD31 was relatively reduced in the glomeruli of *db/db* mice compared to control mice, the colocalization of NLRP3 with CD31 was significantly increased, indicating increased glomerular endothelial NLRP3 expression. Interestingly, overexpression of ELA attenuated glomerular endothelial NLRP3 expression (Fig. 3B).

### Exogenous ELA Treatment Alleviated Glucose-Stressed Inflammatory Actions by Blocking NLRP3 Inflammasome Activation *in vitro*

**Fig. 4:**
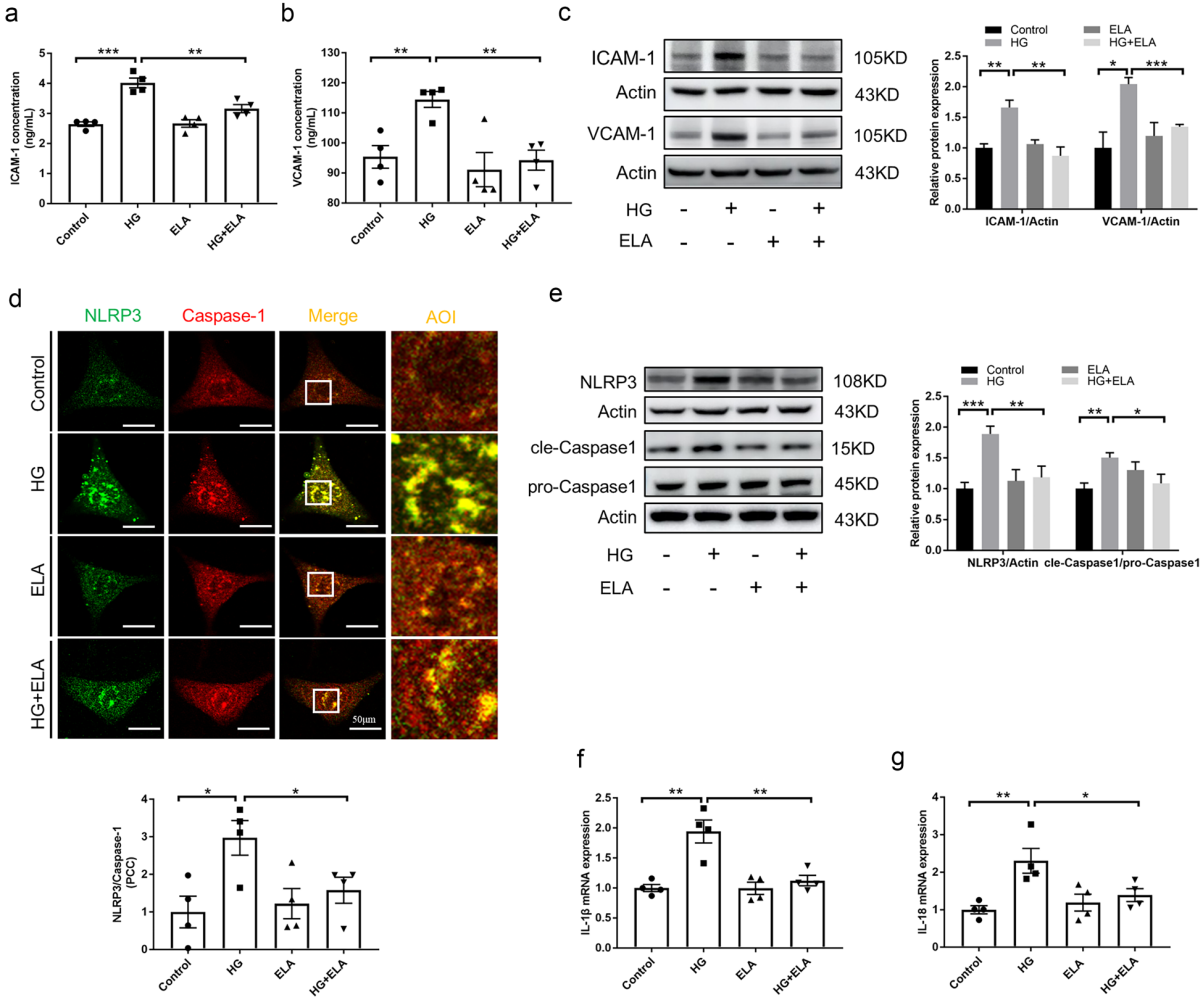
ELA pretreatment attenuates HG-induced inflammatory injury *in vitro*. **A**, **B** Production of secreted ICAM-1 and VCAM-1 (*N* = 5). **C** Representative immunoblots of ICAM-1 and VCAM-1 and the summarized intensities of blots (*N* = 4). **D** NLRP3 (green)/Caspase-1 (red) was identified by confocal microscopy to determine the formation of inflammasomes (*N* = 4). **E** Representative Western blot and the summarized data showing the expression of NLRP3 (*N* = 6) and cle-Caspase 1 (*N* = 6). **F**–**G** mRNA levels of IL-1β and IL-18 (*N* = 5). *P < 0.05, **P < 0.01, ***P < 0.001.

To further investigate the role of ELA in HG-treated GECs, a series of tests were performed with or without ELA peptide pretreatment. We first determined the effects of ELA on adhesive molecules ICAM-1 and VCAM-1 excretion using a commercial kit. The results showed that ELA pretreatment significantly attenuated the elevated excretion of ICAM-1 and VCAM-1 in HG-treated GECs (Fig. 4A–B). Similarly, the protein expression of ICAM-1 and VCAM-1 in GECs, induced by HG stimulation, was significantly reduced by ELA pretreatment (Fig. 4C). Next, the formation and activation of the NLRP3 inflammasome were assessed in HG-treated GECs with or without ELA pretreatment. As shown in Fig. 4D, HG treatment enhanced the colocalization (yellow spots) of NLRP3 (green spots) and Caspase-1 (red spots) compared to the control group, indicating NLRP3 inflammasome formation in GECs. However, this strengthened colocalization could be blocked by ELA treatment. Consistently, ELA pretreatment alleviated the increased production of NLRP3 and cleaved Caspase-1 induced by HG stimulation in GECs (Fig. 4E). Additionally, ELA pretreatment attenuated the increased mRNA production of IL-1β and IL-18 in HG-treated GECs (Fig. 4F–G). These findings suggest that exogenous ELA pretreatment improved HG-induced inflammatory processes by blocking NLRP3 inflammasome activation in cultured GECs.

### ELA Inhibited NLRP3 Inflammasome Activation in an AMPK-Dependent Manner in Glucose-Stressed GECs

**Fig. 5:**
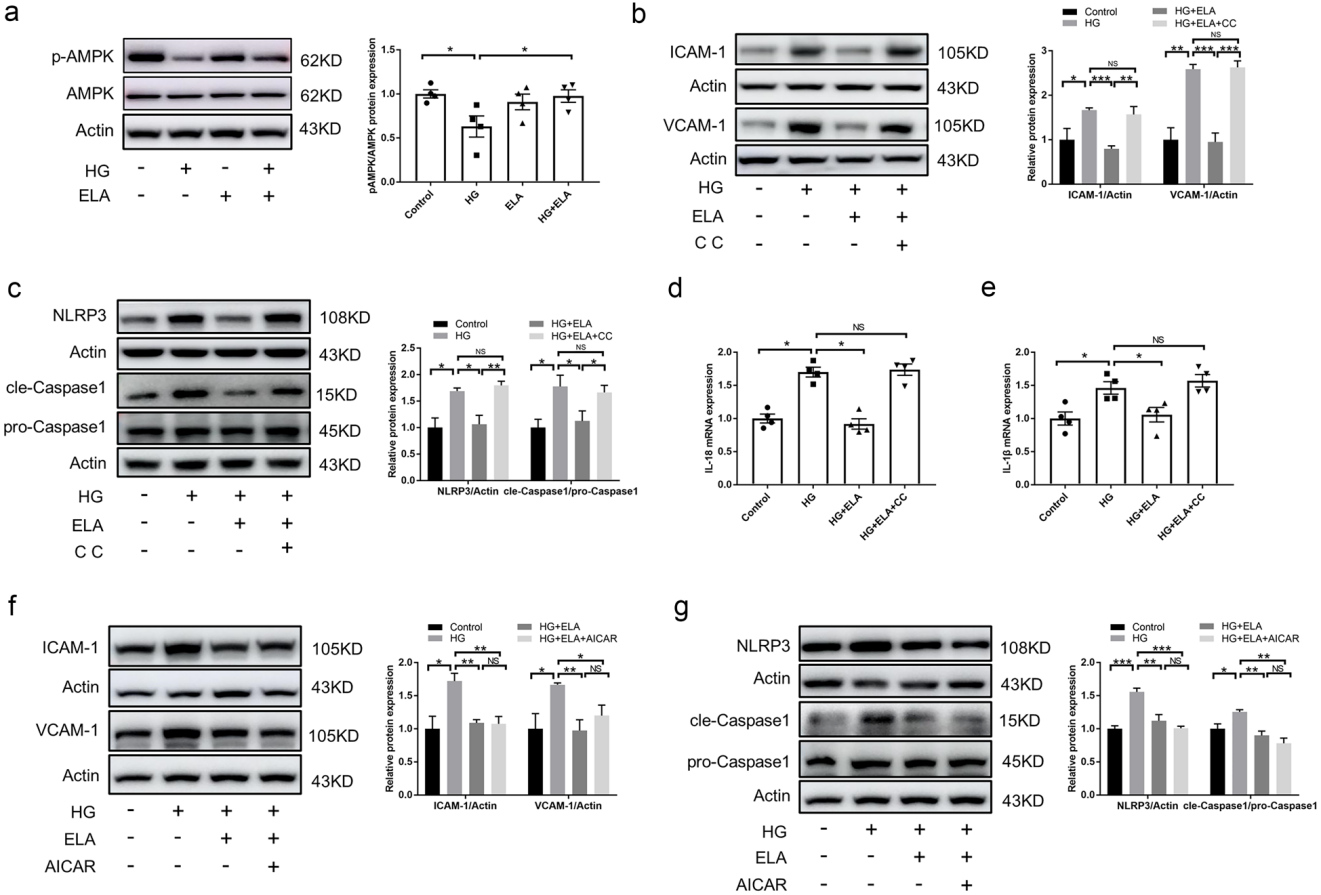
ELA alleviates GECs injury through the AMPK/NLRP3 pathway *in vitro*. **A** Representative Western blot and summarized data showing the expression of p-AMPK in HG-treated GECs with or without ELA pretreatment (*N* = 4). **B**, **C** Representative Western blot and summarized data showing the expression of ICAM-1, VCAM-1, NLRP3, and cle-Caspase 1 in HG-treated GECs with or without ELA or Compound C pretreatment (*N* = 4). **D**, **E** mRNA levels of IL-18 and IL-1β in HG-treated GECs in different groups (*N* = 5). **F**, **G** Representative Western blot and summarized data showing the expression of ICAM-1, VCAM-1, NLRP3, and cle-Caspase 1 in HG-treated GECs with or without ELA or AICAR pretreatment (*N* = 4). *P < 0.05, **P < 0.01, ***P < 0.001.

Previous studies have shown that AMPK activation can alleviate NLRP3 activation in individuals with diabetes [31, 32]. To further explore whether AMPK phosphorylation is involved in the anti-inflammatory effects of ELA, we assessed AMPK activity in HG-treated GECs with or without ELA pretreatment. It was observed that HG stimulation led to a decrease in the p-AMPK/AMPK ratio, which was ameliorated by exogenous ELA pretreatment *in vitro* (Fig. 5A). To investigate whether the anti-inflammatory effects of ELA are mediated through AMPK, we used the AMPK inhibitor Compound C or AMPK siRNA to verify the underlying mechanism. In HG-treated GECs, ELA significantly attenuated the production of ICAM-1 and VCAM-1, as well as the expression of NLRP3 and cleaved Caspase-1. However, pretreatment with Compound C partially reversed the anti-inflammatory effects of ELA (Fig. 5B–C). Consistently, ELA treatment significantly prevented HG-induced IL-18 and IL-1β mRNA expression, while Compound C pretreatment diminished the effects of ELA in GECs (Fig. 5D–E). In addition, genetic inhibition of AMPK via AMPK siRNA was also performed in GECs (Fig. S4A). As shown in Fig. S4B, the expression of NLRP3 and cleaved Caspase-1 in the ELA + siAMPK group was increased compared with the ELA + siNC group. Moreover, we examined the production of ICAM-1, VCAM-1, NLRP3, and cleaved Caspase-1 in GECs with or without the AMPK activator AICAR treatment. The results showed that HG-induced elevation of ICAM-1, VCAM-1, NLRP3, and cleaved Caspase-1 expression was attenuated in both the HG + ELA group and the HG + ELA + AICAR group. However, there were no significant differences between these two groups (Fig. 5F–G). We also performed another selective AMPK activator A769662. Similarly, there were no significant differences between the HG + ELA group and the HG + ELA + A769662 group on the expression of NLRP3 and cleaved Caspase-1 (Fig. S4C). Taken together, these results demonstrate that ELA exerts its beneficial effects on HG-induced GEC injury by regulating the AMPK/NLRP3 pathway.

### ELA-Exerted Effects on the Regulation of AMPK/NLRP3 Pathway Are Dependent on APJ

**Fig. 6:**
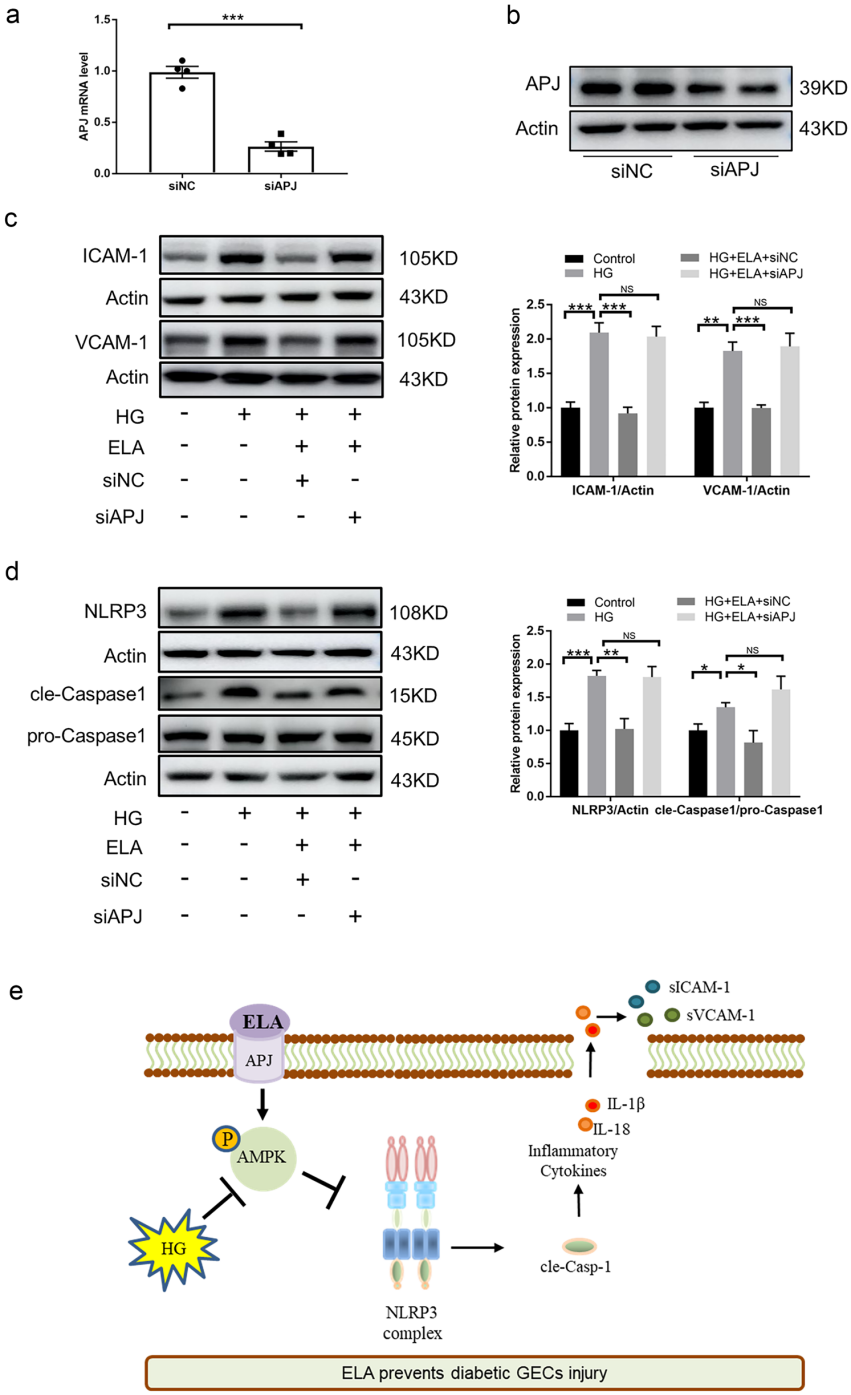
The ELA/APJ axis regulates the AMPK/NLRP3 pathway in glucose-stressed GECs *in vitro*. **A**, **B** APJ knockdown was conducted by the transfection of lentivirus in GECs and confirmed by RT-PCR and Western blot. **C**, **D** Representative Western blot and summarized data showing the expression of ICAM-1, VCAM-1, NLRP3, and cle-Caspase 1 in APJKD cells (*N* = 4). **E** Potential mechanism of ELA on diabetic glomerular endothelial injury. In DKD or HG-treated GECs, ELA/APJ axis improves AMPK activity, blocks NLRP3 inflammasome activation, and attenuates inflammatory cytokines and adhesive molecules. *P < 0.05, **P < 0.01, ***P < 0.001.

ELA was reported to be the second endogenous ligand for the apelin receptor. Thus, we explored whether ELA depended on its receptor APJ to regulate the AMPK/NLRP3 pathway in glucose-stressed GECs. To explore this, we performed APJ knockdown (KD) experiments in GECs and confirmed the efficiency of APJ knockdown through real-time PCR and immunoblotting (Fig. 6A–B). Interestingly, in the APJ KD cells, the HG-induced elevation of ICAM-1 and VCAM-1 expression could not be suppressed by ELA (Fig. 6C). Furthermore, ELA had no significant effects on NLRP3 and cleaved Caspase-1 expression in APJ KD cells (Fig. 6D). In addition, ELA exerted no effects on the p-AMPK/AMPK ratio in APJ KD cells (Fig. S5). These results confirm that the anti-inflammatory effects of ELA in GECs may rely on the presence of its receptor APJ, suggesting the involvement of the ELA/APJ axis in the regulation of the AMPK/NLRP3 pathway. The potential molecular mechanisms of ELA are summarized in Fig. 6E.

## DISCUSSION

This present study explored the potential beneficial effects of ELA on GECs under hyperglycemic conditions and the underlying mechanisms. By investigating the triggering mechanisms of glomerular endothelial injury caused by elevated glucose levels, we aimed to enhance our understanding of DKD and provide valuable insights for the development of new therapeutic strategies.

Previous studies have indicated that serum ELA levels are lower in diabetic patients compared to healthy individuals, and decreased ELA levels are associated with advanced albuminuria and kidney damage in diabetic patients with microalbuminuria [33]. In this present study, we observed significantly decreased ELA mRNA levels in the cortex of STZ-induced diabetic mice and glucose-stressed glomerular endothelial cells. To further investigate the role of ELA in DKD, we utilized heterozygous ELA knockout mice, which is considered a useful model. However, it was reported that loss of the *elabela* gene in mice may cause low penetrance embryonic lethality and defects [34]. Thus, we used heterozygous ELA knockout (ELA^+/−^) mice in this work. Compared with wild-type diabetic mice, ELA^+/−^ diabetic mice exhibited elevated serum creatinine and BUN, as well as increased 24-h total urinary albumin. Furthermore, ELA deficiency exacerbated diabetes-induced glomerular morphological damage, thus demonstrating that ELA may exert an important role in DKD. To further confirm this, ELA overexpression lentivirus was transfected into the renal cortex of *db/db* mice. The ELA gene therapy not only alleviated glomerular morphological changes but also restored endothelial marker CD 31 in *db/db* mice. Furthermore, ELA administration attenuated adhesive molecules ICAM-1 and VCAM-1 expression under hyperglycemia. Intriguingly, Xu et al. [17] found that exogenous ELA-32 administration significantly attenuated ICAM-1 and VCAM-1 mRNA expression in high-salt-loaded Dahl salt-sensitive (SS) rats. Collectively, these findings suggest that ELA may play an anti-inflammatory role in diabetic glomerular endothelial injury.

Given that the expression and synthesis of ICAM-1 and VCAM-1 can be upregulated in response to the pro-inflammatory cytokines IL-1β and IL-18 secreted from inflammasomes in various types of renal resident cells [10], we further investigated the upstream signaling pathway through which ELA exerts its effects on the inflammatory process in diabetic glomerular endothelial cells. The NLRP3 inflammasome has been reported to play a crucial role in initiating inflammatory actions and the production of pro-inflammatory cytokines in DKD [35]. Therefore, we first examined the activation of the glomerular endothelial NLRP3 inflammasome in *db/db* mice with or without ELA administration. Herein, ELA administration alleviated the colocalization of NLRP3 with Caspase-1 and the expression of glomerular endothelial NLRP3 protein in *db/db* mice, indicating the blockade of glomerular endothelial NLRP3 inflammasome formation and activation. Similarly, previous studies have demonstrated that apelin, the first ligand of APJ, attenuates NLRP3 inflammasome activation in rodent models of acute lung injury, cardiomyopathy, and subarachnoid hemorrhage [36–38]. Taken together, ELA may inhibit diabetic glomerular endothelial NLRP3 inflammasome activation.

We further investigated the mechanisms via which ELA regulates the NLRP3 inflammasome in diabetic glomerular endothelial cells (GECs). Previous studies have reported that the apelin/APJ axis can activate AMPK pathways through engagement with heterotrimeric G proteins (Gq), promoting glucose utilization and glucose homeostasis [39]. Additionally, the apelin/APJ axis has been shown to inhibit inflammasome formation by activating AMPK in various experimental conditions, including subarachnoid hemorrhage and respiratory disease [38, 40]. Therefore, we investigated the effects of ELA on AMPK activity regulation, particularly in the context of DKD. Our results demonstrated that ELA treatment restored AMPK phosphorylation levels in GECs during hyperglycemia. Furthermore, the AMPK inhibitor, Compound C, reversed the beneficial effects of ELA and upregulated the expression of NLRP3 and inflammatory cytokines. Consistently, we found no significant difference in the cellular inflammatory response between the HG + ELA group with or without AMPK activator treatment. These findings suggest that ELA may reduce NLRP3 inflammasome activation in an AMPK-dependent manner.

Although accumulating evidence elucidated that AMPK phosphorylation acted as the key factor in the negative regulation of diabetes-induced inflammatory response. However, the precise mechanisms of AMPK phosphorylation on negative regulation of NLRP3 inflammasome activation still remain elusive. It has been reported that AMPKα, an upstream kinase of phosphorylated Akt and eNOS, negatively regulates the generation of reactive oxygen species (ROS) induced by free fatty acids in mitochondria [41]. Previous studies suggested that ROS may trigger NLRP3 formation and activation [42]. Furthermore, AMPK activation leads to phosphorylation and degradation of TXNIP, which further suppresses NLRP3 inflammasome activation [43]. Thus, AMPK activation may suppress NLRP3 inflammasome activation via multiple signaling pathways, such as blockade of ROS generation in mitochondria and phosphorylation and degradation of TXNIP.

Furthermore, we investigated whether ELA exerted beneficial effects through its receptor APJ, which is widely expressed in various tissues, including the kidneys, in rodents and humans [44]. Within the kidney, APJ is localized in the renal cortex and vasculature [45]. Previous studies have demonstrated that ELA exerts beneficial roles in hypertension, heart failure, and cardiac damage by binding to its receptor APJ [28, 46, 47]. However, it is still unclear whether ELA exerts its effects on kidney resident cells through APJ. Chen et al. [48] found that higher concentrations of ELA peptides (0.3 and 3 mM) could induce APJ endocytosis in cultured renal tubular cells, whereas lower concentrations of ELA peptides did not exert such an effect. Our results showed that HG-induced elevation of adhesive molecules and inflammatory cytokines could not be suppressed by ELA pretreatment in the APJ knockdown cells, indicating that ELA’s protective effects and regulation of the AMPK/NLRP3 pathway in glomerular endothelial cells may depend on its receptor APJ. The diverse phenomena observed may be due to different kidney resident cells and different dosages of ELA, which require further exploration and investigation.

Although our present work verified the value of ELA in a novel mechanism mediated via the suppression of the AMPK/NLRP3 pathway, there were limitations that should be clarified. First, we could not directly detect ELA protein expression due to the lack of commercial and specific antibodies for ELA. Second, although ELA is primarily expressed in the endothelium system, ELA may exert its protective effects on different resident cells in DKD through cell-to-cell crosstalk. However, our study focused only on the beneficial effects of ELA in glomerular endothelial cells. Therefore, future studies should investigate the roles of ELA in other cell types relevant to DKD to provide a more comprehensive understanding of its mechanisms of action.

## CONCLUSIONS

In conclusion, our study provides clear evidence that the ELA/APJ axis exerts beneficial effects on diabetic glomerular endothelial injury by regulating the AMPK/NLRP3 signaling pathway, highlighting the potential therapeutic implications of modulating this signaling pathway for the treatment of DKD.

### Supplementary Information

Below is the link to the electronic supplementary material.

Supplementary file1 (DOCX 13.1 KB)

Supplementary file2 (DOCX 949 KB)

## Data Availability

The dataset supporting the conclusions of this article is included within the article.
