# Supplementary material for: ELABELA/APJ Axis Prevents Diabetic Glomerular Endothelial Injury by Regulating AMPK/NLRP3 Pathway
Source: Inflammation. 2023 Aug 4;46(6):2343–58. doi: 10.1007/s10753-023-01882-7 (PMC10673989; doi:10.1007/s10753-023-01882-7)
Supplement: Supplementary file 1 — Supplementary file1 (DOCX 13.1 KB) [file 10753_2023_1882_MOESM1_ESM.docx]

**Table 1. Primers used in this work.**

|  | Sequences (5' to 3') |  |
| --- | --- | --- |
|  | sense | anti-sense |
| Rat-ELA | GCGATGAGTCTCCTTTTTATCACG | TGGGAAGGGCACTCGAGAAT |
| Rat-IL-1β | TCGGCCAAGACAGGTCGCTCA | TGGTTGCCCATCAGAGGCAAGG |
| Rat-IL-18 | GGACTGGCTGTGACCCTATC | TGTGTCCTGGCACACGTTTT |
| Rat-GAPDH | GTCTTCACTACCATGGAGAAGG | TCATGGATGACCTTGGCCAG |
| Mouse-ELA | CAGAAACCAGTTAACTTTCCCAGG | TGGGAAGGGCACTCGAGAAT |
| Mouse-GAPDH | CTCCCACTCTTCCACCTTCG | CCACCACCCTGTTGCTGTAG |
