# Supplementary material for: ELABELA/APJ Axis Prevents Diabetic Glomerular Endothelial Injury by Regulating AMPK/NLRP3 Pathway
Source: Inflammation. 2023 Aug 4;46(6):2343–58. doi: 10.1007/s10753-023-01882-7 (PMC10673989; doi:10.1007/s10753-023-01882-7)
Supplement: Supplementary file 2 — Supplementary file2 (DOCX 949 KB) [file 10753_2023_1882_MOESM2_ESM.docx]

**Supplementary figure 1**


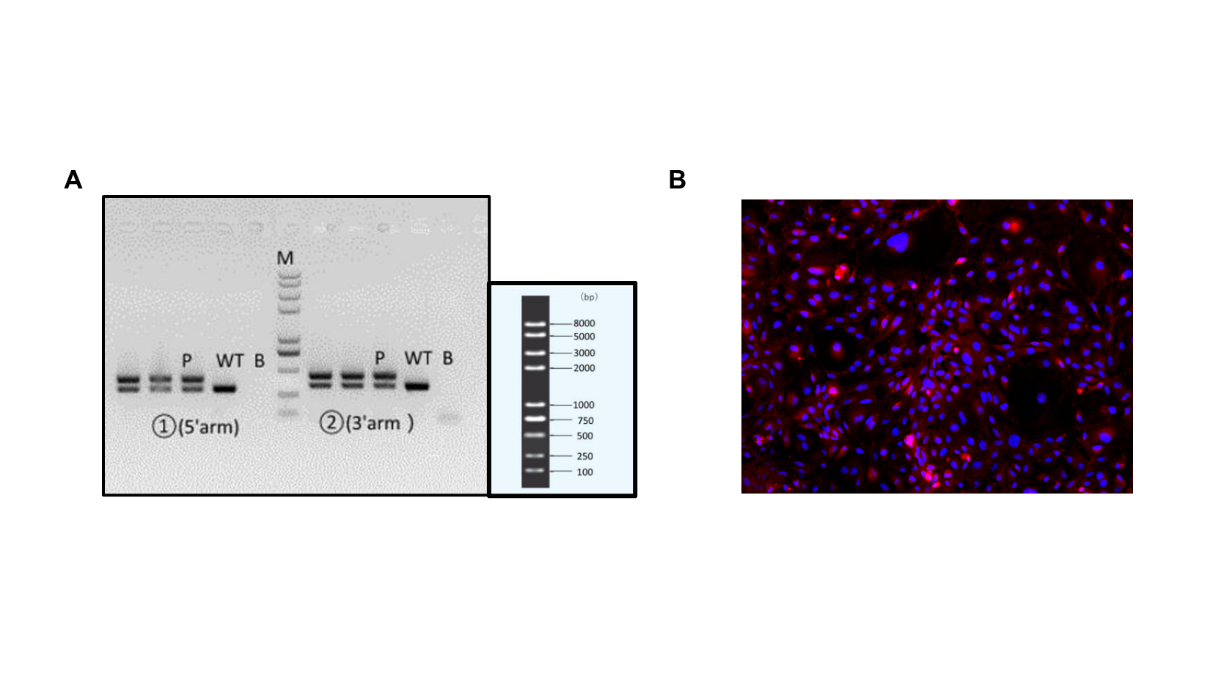


(**A**)The genotyping figure of heterozygous ELA knockout mice. (**B**) Identification of rat glomerular endothelial cells via positive staining with CD31. Magnification: 200X.

**Supplementary figure 2**


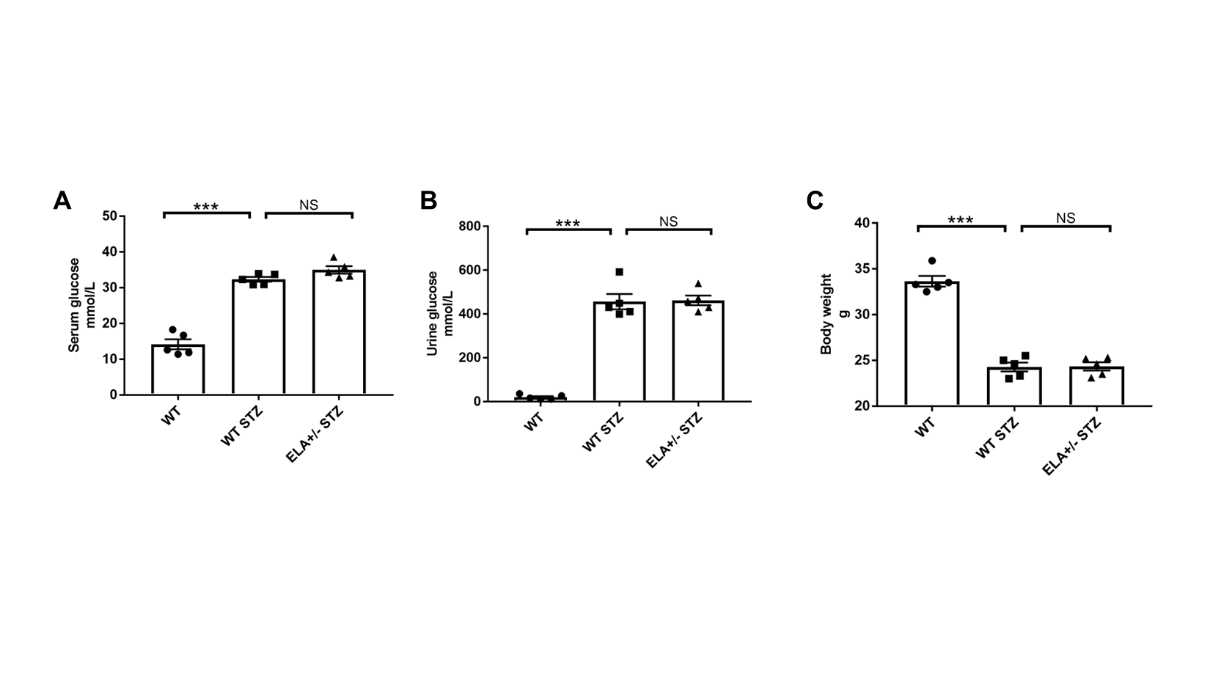


**(A-C)** Serum glucose, urinary glucose and body weight in different groups (N=5). ***P<0.001; NS, no sense.

**Supplementary figure 3**


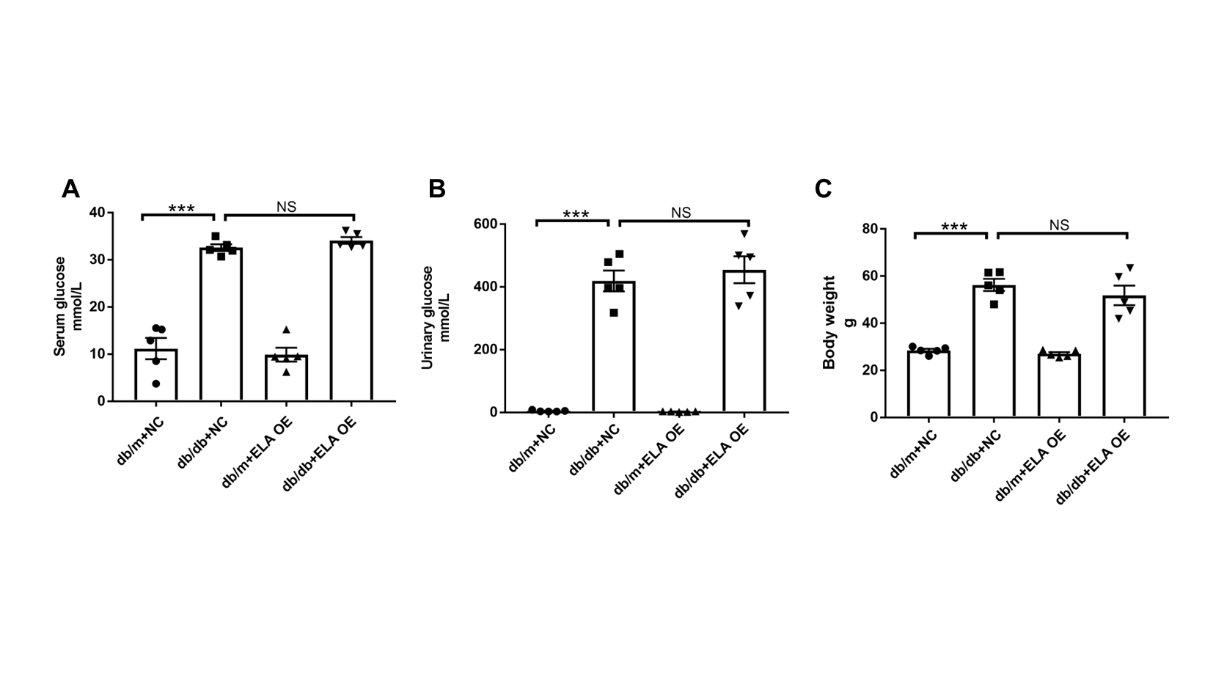


**(A-C)** Serum glucose, urinary glucose and body weight in different groups (N=5). ***P<0.001; NS, no sense.

**Supplementary figure 4**


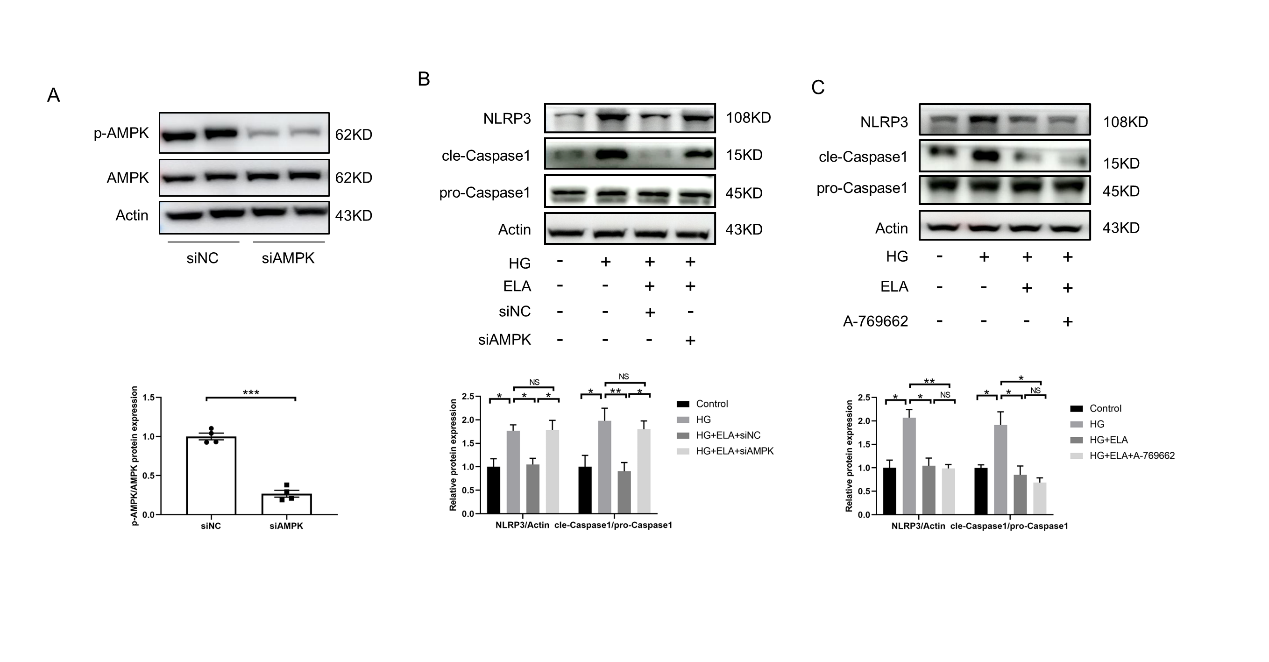


**(A)** Representative Western blot and summarized data showing the expression of p-AMPK in siNC and siAMPK group (N=4). **(B)** Representative Western blot and summarized data showing the expression of NLRP3 and cleaved Caspase-1 in HG-treated GECs with or without siAMPK pretreatment (N=4). **(C)** Representative Western blot and summarized data showing the expression of NLRP3 and cleaved Caspase-1 in HG-treated GECs with or without ELA or A-769662 pretreatment (N=3). *P<0.05; **P<0.01; NS, no sense.

**Supplementary figure 5**


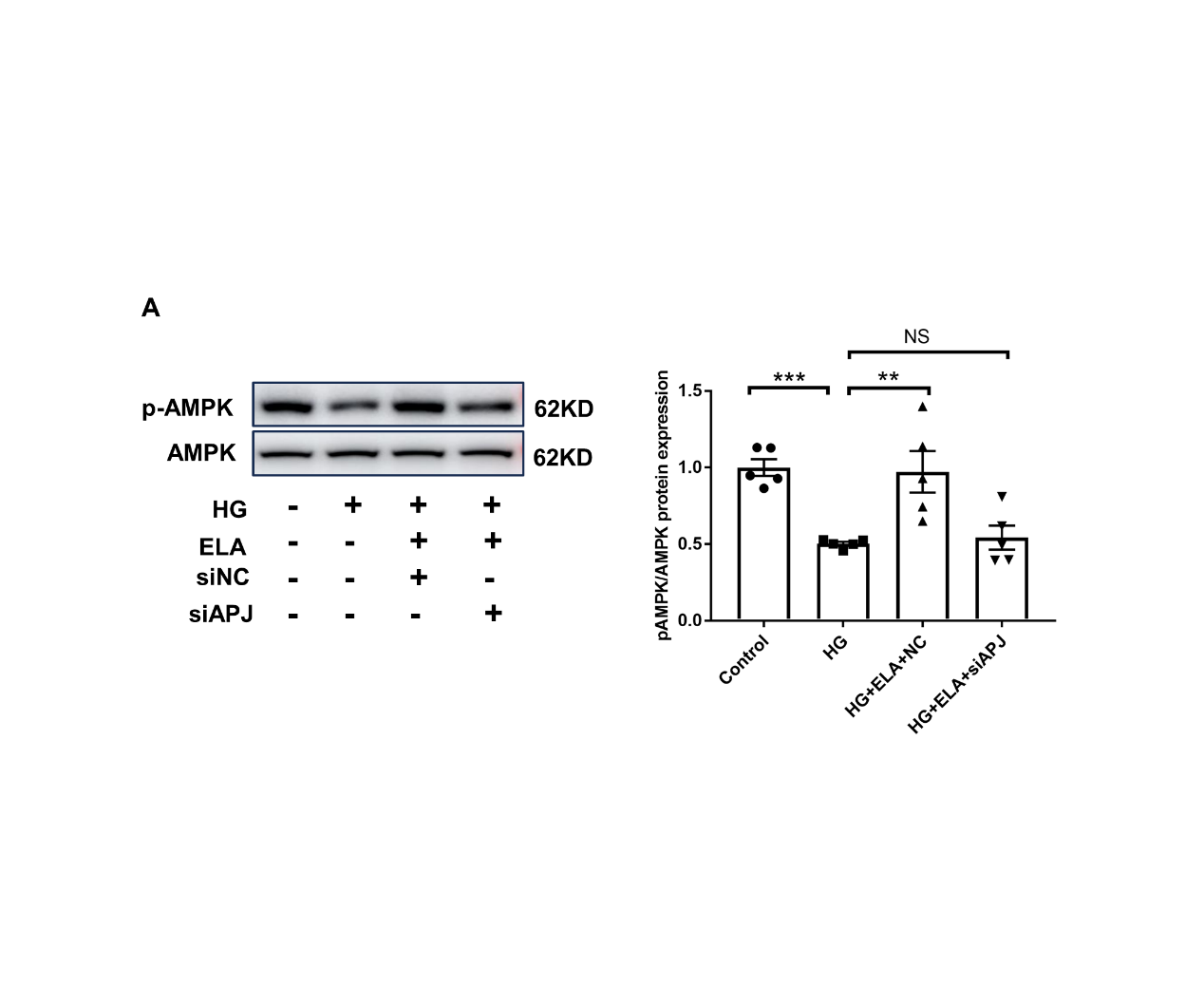


**(A)** Representative Western blot and summarized data showing the expression of p-AMPK in different groups (N=5). **P<0.01; ***P<0.001; NS, no sense.
